# Inhibition of Human Immunodeficiency Virus Type 1 Entry by a Keggin Polyoxometalate

**DOI:** 10.3390/v10050265

**Published:** 2018-05-16

**Authors:** Xiaoli Wang, Jiao Wang, Wenmei Zhang, Boye Li, Ying Zhu, Qin Hu, Yishu Yang, Xiaoguang Zhang, Hong Yan, Yi Zeng

**Affiliations:** 1 College of Life Science and Bioengineering, Beijing University of Technology, Beijing 100124, China; wangxiaoli@bjut.edu.cn (X.W.); zwm1436271046@sina.com (W.Z.); liboye1994@sina.com (B.L.); zhuying19901101@163.com (Y.Z.); yishu-y@bjut.edu.cn (Y.Y.); hongyan@bjut.edu.cn (H.Y.); 2 National Institute for Viral Disease Control and Prevention, Chinese Center for Disease Control and Prevention, Beijing 102206, China; wangjiao1018@hotmail.com

**Keywords:** human immunodeficiency virus type 1, Keggin polyoxometalate, entry inhibition, CD4, gp41 NHR

## Abstract

Here, we report the anti-human immunodeficiency virus (HIV) potency and underlying mechanisms of a Keggin polyoxometalate (PT-1, K_6_HPTi_2_W_10_O_40_). Our findings showed that PT-1 exhibited highly potent effects against a diverse group of HIV type 1 (HIV-1) strains and displayed low cytotoxicity and genotoxicity. The time-addition assay revealed that PT-1 acted at an early stage of infection, and these findings were supported by the observation that PT-1 had more potency against Env-pseudotyped virus than vesicular stomatitis virus glycoprotein (VSVG) pseudotyped virus. Surface plasmon resonance binding assays and flow cytometry analysis showed that PT-1 blocked the gp120 binding site in the CD4 receptor. Moreover, PT-1 bound directly to gp41 NHR (N36 peptide), thereby interrupting the core bundle formation of gp41. In conclusion, our data suggested that PT-1 may be developed as a new anti-HIV-1 agent through its effects on entry inhibition.

## 1. Introduction

Human immunodeficiency virus type 1 (HIV-1) is the major cause of acquired immunodeficiency syndrome (AIDS). It has infected more than 36 million people worldwide by the end of 2015, according to the United Nations Joint Programme on HIV/AIDS (UNAIDS) reports. Significant success has been achieved in HIV-1 treatment since the introduction of highly active antiretroviral therapy (HAART) in the 1990s [1]. Current antiretroviral therapy, with three to four anti-HIV-1 drugs used in combination, targets multiple steps in the HIV-1 life cycle, including the viral reverse transcriptase, integrase, protease, viral entry, and fusion.

Viral entry and fusion inhibitors are some of the most attractive agents in HIV-1 therapy [2]. In the last several decades, great progress has been made in the understanding of the mechanisms of HIV-1 entry into host cells. It involves the binding of viral glycoprotein gp120 to the cellular CD4 receptor, and subsequent reconfiguration of gp120 to allow the interaction of gp120 and a cellular coreceptor, i.e., CCR5 or CXCR4. The interaction was followed by additional conformational changes in the viral envelope, resulting in exposure of gp41 transmembrane protein and ultimately driving the fusion of the viral envelope with the host membrane [3,4,5,6,7,8]. To date, the CCR5 antagonists maraviroc (MVC) and the fusion inhibitor targeting gp41, T20 (enfuvirtide) have been approved for clinical use, and many other inhibitors targeting various stages of viral entry are still in development [9,10].

Polyoxometalates (POMs) are early transition metal clusters linked to oxygen and other atoms. POMs exhibit a great diversity in structure and are widely used as catalysts in material sciences and medicine. Two basic structures of POMs, Keggin and Dawson, have attracted attention owing to their antiviral properties, and these POMs have been shown to have activity against a broad range of DNA and RNA viruses, including influenza A and B, severe acute respiratory syndrome coronavirus (SARS-V), respiratory syncytial virus and dengue virus [11,12,13,14,15]. The POM known as ammonium-21-tungsto-9-antimoniate (HPA-23) ended with an HIV/AIDS clinical trial in the 1980s. This compound failed due to unacceptable adverse effects and minor benefits [16,17,18]. Since then, several groups have synthesized and identified a number of new generations of POMs with improved potency and reduced toxicity [18,19,20,21,22,23,24,25,26,27], although the mechanisms remain unclear. We have previously identified the anti-HIV activity of a number of Keggin POMs and derivatives [28]. In the present work, we evaluated the potency and mechanism of action of a Keggin polyoxometalate PT-1 (K_6_HPTi_2_W_10_O_40_, Cambridge Crystallographic Data Centre, CCDC: 1440344) in the prevention of HIV-1 entry.

## 2. Materials and Methods

### 2.1. Cells, Viruses and Compounds

TZM-bl cells were obtained from the National Institutes of Health AIDS Reagent Repository and cultured in Dulbecco’s modified Eagle’s medium (DMEM; Gibco, Shanghai, China) supplemented with 10% (*v*/*v*) fetal bovine serum (FBS; Hyclone, Shanghai, China), 200 µg/mL l-glutamine and 100 U/mL penicillin-streptomycin. HEK-293T cells and the human T-cell line MT4 was purchased from American Type Culture Collection (ATCC) and maintained in RPMI 1640 (Gibco) supplemented with 10% (*v*/*v*) fetal bovine serum, 200 µg/mL l-glutamine and 100 U/mL penicillin-streptomycin. Peripheral blood mononuclear cells (PBMC) were isolated from three healthy donors and cultured in complete RPMI medium (10% fetal bovine serum, 200 µg/mL l-glutamine, 100 U/mL penicillin-streptomycin, and 25 ng/mL interleukin-2 (IL-2)). The HIV Env clones Q842env.d16 (NIH#11886), Q259env.w6 (NIH#11887), TRO.11 (NIH#11023), REJO4541.67 (NIH#11035), SC422661.8 (NIH#11058), Du422.1 (NIH#11308), QA790.204I.ENV.A4 (NIH#11901), QA790.204I.ENV.C1 (NIH#11902), and backbone vector pSG3∆env (NIH#11051) were obtained from the National Institutes of Health AIDS Reagent Repository. BJOX00200 env (NIH#12655), CH119 env (NIH#12659), CE1176 env (NIH#12657), and CNE8 env (NIH#12653) were kindly provided by Dr. Lei Yu (Shenzhen CDC, China). HIV-1 NL4-3, HXB2, and BH10 strains were kindly gifted from Institute of Biophysics, Chinese Academy of Sciences. pCMV-VSV-G Envelope Vector was kindly gifted by Dr. Rui Pedro Galao (King’s College London, London, UK). PT-1(K_6_HPTi_2_W_10_O_40_) was synthesized by Dr. Yan Hong as previously described [28,29]. The structure information of PT-1 was deposited at the Cambridge Crystallographic Data Centre (CCDC: 1440344). The aqueous stock solution of PT-1 (3.5 mM) was stored at −20 °C.

### 2.2. Cell Viability

The cell counting kit-8 assay (CCK-8 assay, Dojindo, Shanghai, China) was performed to determine the cell viability of TZM-bl, HEK-293T and MT4 cells after PT-1 treatment. The cells seeded in a 96-well plate were treated with a serial dilution of PT-1 ranging from 3.5 to 70 µM for 48 h. At the end of experiment, cells were incubated with CCK-8 solution for 1 h and measured at 450 nm using the Enspire^®^ Multi-mode Plate Reader (PerkinElmer, Waltham, MI, USA). The cytotoxicity (CC_50_ values) was calculated as 50% cytotoxic concentration using GraphPad Prism 6 software (version 6.01, GraphPad Software Inc., La Jolla, CA, USA). The selectivity index was calculated by CC_50_/IC_50_ (The half maximal inhibitory concentration).

### 2.3. In Vivo Mouse Micronucleus Test

Micronucleus tests were conducted as previously described [30]. Briefly, 25 male Kunming mice (6-weeks old, 18–20 g) were obtained from Beijing Vital River Laboratory Animal Technology Co., Ltd., Beijing, China. Animals were acclimated for a week and randomly allocated into the following groups with five mice each: vehicle control (normal saline), 200 mg/kg PT-1 (low-dose), 600 mg/kg PT-1 (mid-dose), 1800 mg/kg PT-1 (high-dose) and positive control (cyclophosphamide, CP, 40 mg/kg). Mice were administrated intragastrically with PT-1/normal saline or intraperitoneally with CP once a day for three days. Mice were sacrificed 48 h post the last administration, then the bilateral femur was separated, bone marrow cells were collected from the aforementioned femur in fetal bovine serum, centrifuged at 1000× *g* for 5 min, and smeared onto slides. Slides were air-dried and fixed by submerging in methanol for 15 min, followed by Giemsa staining for 15 min. Slides were then randomly coded and counted under ×1000 magnification. Data were expressed as the ratio of immature erythrocytes (polychromatic erythrocytes, PCE) to total erythrocytes (PCE + normochromatic erythrocytes, NCE) by counting at least 200 erythrocytes and the number of micronucleated (MN) PCE in 1000 PCEs. The study was carried out with approval of the Animal Ethical Committee of China Academy of Chinese Medical Sciences (approval code: 2014059).

### 2.4. Single-Round Replication Assay

The anti-HIV-1 activity of PT-1 was performed using the TZM-bl assay [31]. The HIV-1 Env-pseudotyped viruses were prepared by co-transfection HEK-293T cells with HIV-1 pREJO4541.67 Env expression plasmid and pSG3∆env using FuGENE transfection reagent (Promega, Madison, WI, USA). The recombinant viruses were collected from cell supernatant after centrifugation at 2500 rpm for 5 min and stored in liquid nitrogen. The titers of HIV-1 Env-pseudotyped viruses were examined by the 50% tissue culture infective dose (TCID_50_) assay using Reed-Muench method. Late, TZM-bl cells were seeded in the 96-well plate and infected with 1000 TCID_50_/mL virus diluted in diethylaminoethanol (DEAE) dextran (Sigma, Shanghai, China) in the presence of PT-1. Two days after infection, the infectivity of viruses was evaluated using the Bright-Glo luciferase assay kit (Promega) under the manufacturer’s instructions. The luciferase activity was analyzed using the Enspire^®^ Multi-mode Plate Reader (PerkinElmer) and expressed in terms of relative luciferase units (RLUs). The half maximal inhibitory concentration (IC_50_) was calculated as a 50% inhibitory concentration using GraphPad Prism 6 software.

To assess the activity of PT-1 on HIV-1 virus entry, vesicular stomatitis virus glycoprotein (VSVG) pseudotyped viruses were produced by co-transfected HEK-293T cells with pCMV-VSVG and pSG3∆env plasmids. Virus titers were determined by the TCID_50_ assay. The TZM-bl cells were then infected with either 1000 TCID_50_/ml Env-pseudotyped viruses or the equal amount of VSVG-pseudotyped viruses. After the addition of indicated doses of PT-1 and incubation for 48 h, the luciferase activity was measured (Bright-Glo luciferase assay kit, Promega) to compare the difference between Env and VSVG-mediated virus entry inhibition.

### 2.5. Wide-Type HIV-1 Replication Assay

The human T-cell line MT4 cells were infected with HIV-1_NL4-3_, HIV-1_HXB2_ or HIV-1_BH10_ (1000 TCID_50_/mL) in the presence of the indicated concentration of PT-1 or positive control zidovudine (AZT). Two-hour post-infection, cells were washed with phosphate-buffered saline (PBS) and transferred to a 96-well plate. The cultures were incubated with medium replacement at day 3 and analyzed at day 5. The culture supernatants were harvested and quantified using a p24 enzyme-linked immunosorbent assay (ELISA) (Vironostika HIV-1 antigen ELISA kit, Biomerieux, Lyon, France).

### 2.6. Inhibition of HIV-1 Replication in Peripheral Blood Mononuclear Cells

Peripheral blood mononuclear cells were isolated from three healthy blood donors using a Ficoll gradient (GE Healthcare, Chicago, IL, USA). PBMC were pre-stimulated with ConA (5 µg/mL; Sigma) at a density of 1 × 10^6^ cells/mL for 2 days, then the activated PBMC were washed twice with PBS and maintained in a culture medium containing human recombinant IL-2 (25 ng/mL; PeproTech Inc., Suzhou, China) during viral infection. PBMC (1 × 10^5^/well) were incubated with HIV-1_NL4-3_ (5 × 10^4^ TCID_50_) and increasing concentrations of PT-1 (0.007, 0.027, 0.11, 0.44, 1.76, 3.52, and 7.04 μM) or AZT (0.004, 0.037, 0.37, and 3.74 μM) for 3 h at 37 °C. Infected cells were washed twice and added to 96-well plates containing the fresh PT-1 or AZT solution. On days 3, 5, 7 post-infection, half of the medium was removed and replaced with fresh medium containing PT-1 or AZT. Supernatants were collected at days 3, 5, 7, and 9 after infection and tested for p24 antigen (Vironostika HIV-1 antigen ELISA kit). AZT was used as positive control.

### 2.7. Time-of-Addition Assay

A time-of-addition study was conducted using a single-round replication assay. The TZM-bl cells were infected with HIV-Env-pseudotyped viruses (REJO4541.67) for 2 h, then the cells were washed extensively to remove supernatant, and the fresh product was added to continue the experiment. PT-1 (1.7 μM) was added to the culture medium at 0 h, 0.25 h, 0.5 h, 1 h, 2 h, 6 h, 9 h and 24 h after infection, and maraviroc (MVC, 1 μM, CCR5 receptor antagonist), azidothymidine (AZT, 1 μM, Reverse-transcriptase inhibitor), raltegravir (RAL, 1 μM, integrase inhibitor), and T20 (1 μM, fusion inhibitor) severed as controls. At the end of the experiments, the inhibition of viral replication was measured by luciferase activity.

### 2.8. Flow Cytometry Assay

The interaction of PT-1 and CD4 receptor was assessed by flow cytometry (FACS) analysis. Briefly, MT-4 cells were harvested and incubated with or without PT-1 (0.7, 3.5, and 17.6 μM) or gp120 (0.84 μM, positive control) for 30 min. Cell were then washed with FACS buffer and stained with PE anti-human CD4 antibody (BD Pharmingen, Shanghai, China) for 30 min on ice. After washing, the cells were resuspended in the FACS buffer for FACS analysis (BD FACS Calibur flow cytometer, BD Biosciences, Franklin Lakes, NJ, USA).

The cell apoptosis of PBMCs were detected using Annexin V, FITC Apoptosis Detection Kit (Dojindo, Tokyo, Japan). PBMCs were isolated and activated with ConA (5 µg/mL, Sigma) for 2 days as described. Pre-stimulated PBMC (1 × 10^5^/well) were then incubated with PT-1 (1.76, 8.80, and 44.00 µM) in fresh RPMI1640 (containing 25 ng/mL IL-2) for another 3 days. On day 5, cells were collected and stained with Annexin-V-FITC and propidium iodide (PI) following manufacturer’s instruction. All data were acquired with a BD FACS Calibur flow cytometer (BD Biosciences, San Jose, CA, USA).

### 2.9. Native Polyacrylamide Gel Electrophoresis

The activity of PT-1 on the formation of HIV-1 gp41 six-helix bundle was measured by native polyacrylamide gel electrophoresis (N-PAGE) assay. In general, 30 μM N-peptide N36 (synthesized by Beijing Scilight Biotechnology Ltd., Beijing, China) was incubated with different concentration of PT-1 at 37 °C for 30 min, then 40 μM C34 peptide was added for another 30 min of incubation, and the samples were mixed with Tris-glycine native sample buffer and loaded onto 18% Tris-glycine gel for electrophoresis at 120 V constant voltage. After 90 min electrophoresis, the gel was stained with Coomassie Blue dyes and imaged with Bio-Rad Gel Doc XR^+^ Imaging System (Bio-Rad, Hercules, CA, USA). AZT was used as a negative control.

### 2.10. Surface Plasmon Resonance Binding Assay

Surface plasmon resonance (SPR) binding assay was performed using BIAcore 3000 (GE Healthcare) [32]. The surface of the CM5 sensor chip was activated by injecting 0.05 M *N*-hydroxysuccimide (NHS) and 0.2 M *N*-ethyl-*N*′-(diethylaminopropyl)-carbodiimide (EDC) at a flow rate of 10 μL/min for 10 min. The N36 peptide (5 μM), CD4 recombinant protein (0.5 μM), and gp120 (0.5 μM) recombinant protein were dissolved in 10 mM acetate buffer (pH 5), respectively and immobilized to chips using amine coupling. The binding sites were then blocked with a 1.0 M ethanolamine-HCl (pH 8.5) injection. In the direct binding assay, PT-1 of diluted serial concentrations was injected over the surface of N36 peptide and CD4 protein at the rate of 10 μL/min for 10 min. In the competition assay, 0.7 μM PT-1 were incubated with 1.1 μM soluble CD4 protein for 15 min prior to injection. Then, 2 μM PT-1, 1.1 μM CD4 and the PT-1/CD4 mixture were injected to the flow cell over the gp120 protein, respectively. In both assays, the unmodified surface was used as a reference surface. The resonance unit value (RU) was measured for data analysis. The equilibrium constant for dissociation (KD) values was determined using the BIAvaluation software (GE Healthcare).

### 2.11. Reverse Transcriptase Assay

The inhibitory effect of PT-1 on reverse transcriptase (RT) was measured using commercial colorimetric assay (Roche, Berlin, Germany) following the manufacturer’s instructions. Briefly, PT-1 (0.7, 3.5, and 17.6 μM) were incubated with 4 ng HIV-1 RT, digoxigenie (DIG) and biotin-labeled DNA solution per reaction tube for 1 h at 37 °C. The mixture was then incubated with a streptavidin coated surface of a microplate for 1 h at 37 °C. After extensively washing, DIG-labeled DNA was co-incubated with the peroxidase conjugated anti-DIG-POD antibody for 1 h. Later, 200 μL 2,2′-azino-bis(3-ethylbenzothiazoline-6-sulphonic acid (ABTS) substrate was added to each well after washing, and after 30 min of incubation, the absorbance of the cleaved product was measured at OD 405 nm and the percent inhibition was calculated as compared to the no inhibitor control, with nevirapine (NVP) used as a positive control.

### 2.12. Protease Assay

The inhibitory effect of PT-1 on HIV-1 protease was measured by commercial fluorimetric assay (AnaSpec, Fremont, CA, USA) according to the manufacturer’s instructions. In brief, PT-1 was first incubated with HIV-1 protease in an assay buffer for 15 min, then the mixture was incubated with a HIV-1 protease substrate solution for 60 min at room temperature. The fluorescence intensity was measured at Ex/Em = 490 nm/520 nm. Pepstatin A was used as a positive control.

### 2.13. HIV-1 Integrase 3′ Processing Assay

The inhibitory effect of PT-1 on HIV-1 integrase 3′ processing activity was performed using the fluorescence resonance energy transfer assay as previously described [33]. The Sso7d fusion integrase was kindly gifted from Liu Wei from Beijing University of Technology. Oligonucleotide substrates were synthesized from SBS Genetech (Beijing, China). The sequences were: 5′ [FAM]–ACTGCTAGAGATTTTCCACGTGGAAAATCTCTAGCAGT-[DABCYL]-3′. The DNA substrates were dissolved in the annealing buffer (10 mM, Tris-HCl, 50 mM NaCl, 1 mM EDTA, Ph = 8.0), denatured for 2 min at 94 °C, and annealed for 2 h by dropping 1 °C every 90 s. The 3′ processing reaction was performed in a black solid-bottom 96-well plate. Briefly, PT-1 was incubated with 2.5 μg integrase in reaction buffer (0.25 M DTT, 200 mM NaCl, 50% glycerol, 40 mM Hepes) for 30 min at 37 °C. Subsequently, 20 pM substrates and 10 mM MnCl_2_ were added to start reaction. After 4 h incubation, the fluorescence intensity was measured at Ex/Em = 485 nm/528 nm by Enspire^®^ Multi-mode Plate Reader. RAL was used as a positive control.

### 2.14. Statistical Analysis

The results are presented as mean ± standard deviation (SD). All the statistical analyses were performed using the GraphPad Prism software. 

## 3. Results

### 3.1. PT-1 Exhibited Potent Antiviral Activity and Low Toxicity

**Figure 1:**
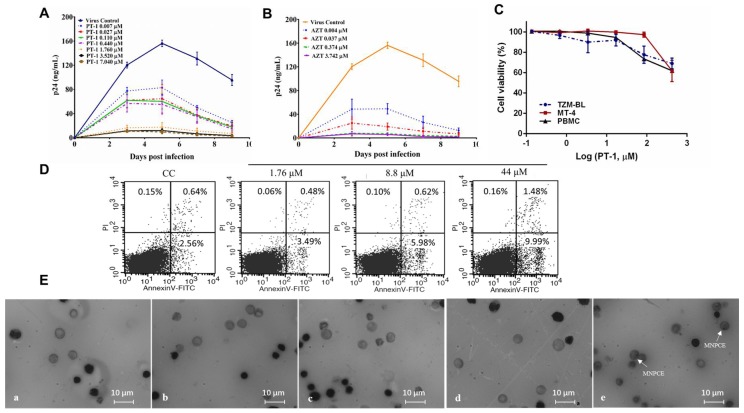
The anti-human immunodeficiency virus (HIV) activity in activated peripheral blood mononuclear cells (PBMCs) and toxicity of the Keggin polyoxometalate PT-1. ConA stimulated PBMCs were incubated with the HIV-1 NL4-3 strain and increasing doses of PT-1(**A**) or zidovudine (AZT) (**B**), HIV-1 replication was evaluated by HIV-1 p24 enzyme-linked immunosorbent assay (ELISA) on days 3, 5, 7, and 9 after infection, and the data were expressed as the means ± standard deviations (SD); (**C**) The cytotoxicity of PT-1 were measured using TZM-bl, MT4, and PBMCs with Cell counting kit -8 (CCK-8) assay; (**D**) Representative plots of PBMC apoptosis measured by Annexin V-FITC/PI staining and flow cytometry (FACS) analysis. Early apoptotic cells (Annexin-V positive-PI negative) and late apoptotic cells (Annexin-V-PI positive) are shown in the lower right and upper right, respectively; (**E**) Representative image of mice bone marrow cells smears from vehicle control (**a**), 200 mg/kg PT-1 (**b**), 600 mg/kg PT-1 (**c**), 1800 mg/kg PT-1 (**d**), and cyclophosphamide positive control group (**e**). Cells were fixed and stained with Giemsa stain, magnification 1000× polychromatic erythrocytes (PCEs) with one or more nuclei (white arrow) scored. All data represent two independent experiments.

PT-1 was evaluated for antiviral activity on 15 laboratory strains using TZM-bl cells or T cells. The corresponding half-maximal inhibitory concentration (IC50) values are summarized in Table 1. Considerable variability in the susceptibilities of virus subtypes to PT-1 was observed, with PT-1 inhibited subtypes B at a relatively higher potency and showed less sensitivity towards subtype C and A in TZM-bl cells. In MT4 cells, the replication of the wide-type virus was significantly inhibited by PT-1 at IC50 ranging from 0.47 to 1.56 µM. To confirm the inhibition in primary T cells, PT-1 was tested using HIV-1 NL4-3 and PBMCs from three healthy donors, respectively. PT-1 exhibited a strong inhibitory effect on PBMCs with IC50 and IC90 (Figure 1A and Table S1), The HIV-1 nucleoside reverse transcriptase inhibitor AZT, serving as a positive control, exhibited broad inhibitory activities against all tested viruses (Figure 1B). In addition to its remarkable high potency, PT-1 showed low cytotoxicity in TZM-bl, MT4 cells and PBMCs, even at a concentration that was 100-fold higher than the effective dose (Figure 1C and Table 1). No significant changes in the percentage of early and late apoptotic cells were seen in PBMCs and MT4 cells after PT-1 treatment (Figure 1D and Figure S1). Furthermore, the genotoxic effect of PT-1 was monitored using mouse bone marrow micronucleus tests (Figure 1E, Table S2), and the data showed that PT-1 at doses up to 1800 mg/kg resulted in no mortality and bone marrow toxicity. Moreover, a serial of PT-1 organic analogs with amino acid modification were tested using TZM-bl assay, and they all exhibited potent anti-HIV activity (Figure S2). Thus, PT-1 was a powerful inhibitor of HIV-1 infection.

### 3.2. PT-1 Inhibited HIV-1 Replication at Early Stage

**Figure 2:**
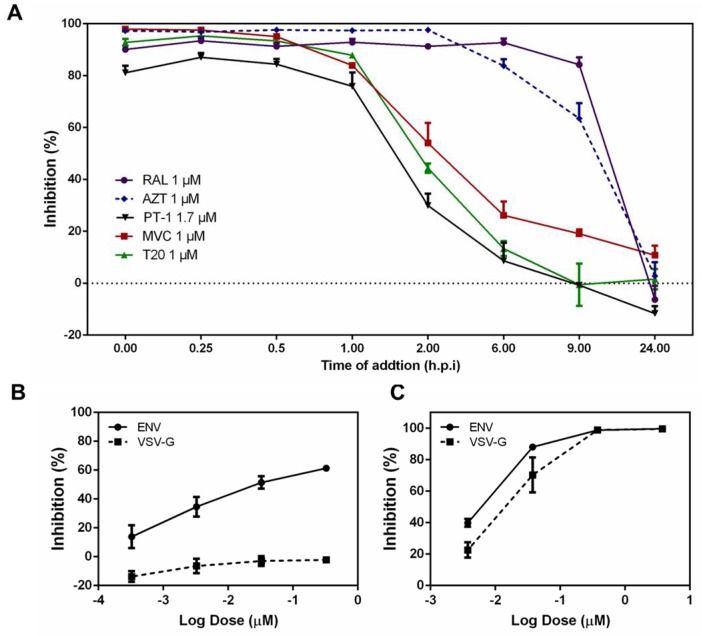
PT-1 inhibited HIV-1 replication at an early stage. (**A**) Time-of-addition studies were conducted using TZM-bl assay. TZM-bl cells were infected with HIV-1 pREJO4541.67 before PT-1 (1.7 μM), Maraviroc (MVC, 1 μM), Azidothymidine (AZT, 1 μM), T20 (1 μM) and raltegravir (RAL, 1 μM) were added upon HIV-1 inoculation (0 h) or at indicated time points. Luciferase activity was determined 48 h after infection. Comparison of the effects of PT-1 (**B**) and AZT (**C**) on the replication of Env and VSV-G pseudovirions. TZM-bl cells were infected with 1000 TCID_50_ (50% tissue culture infective dose)/mL Env (pREJO4541.67) and VSV-G pseudovirions, respectively. After 48 h of treatment with PT-1 or AZT, inhibition was analyzed by luciferase activity assays. All data were performed in triplicate and repeated in three independent experiments.

To assess the mode of action of PT-1, we first conducted time-of-addition assays to thoroughly investigate possible targets of the HIV-1 life-cycle. The HIV-1 reverse transcriptase inhibitor AZT, fusion inhibitor T20, integrase inhibitor raltegravir, and CCR5 inhibitor maraviroc (MVC) served as controls. PT-1 was added to TZM-bl cells at different time points (pre viral adsorption 0 h, virus-cell fusion 0.25, 0.5, and 1 h, reverse transcription 2 h, integration 6 h, transcription 9 h and maturation 24 h) [34]. Cells were collected 48 h post-infection and analyzed for luciferase activity. As illustrated in Figure 2A, the reverse transcriptase inhibitor AZT exhibited potent anti-HIV activity for up to 2 h, whereas RAL began to lose inhibition activity after 8 h. PT-1, CCR5 inhibitor MVC and fusion inhibitor T20 showed inhibition from 0 h to 2 h, indicating that PT-1 inhibited a very early step of virus replication, possibly the viral entry process. We next analyzed the effects of PT-1 on the attachment of HIV-1 Env protein to target cells by comparing Env pseudotyped and VSV-G pseudotyped virus infection using TZM-bl assays. As noted in Figure 2B, inhibition was abolished after replacement of the HIV-1 envelope with VSV-G, although no differences of inhibition were observed in AZT treated cells (Figure 2C), indicating that PT-1 specifically interacted with the HIV-1 glycoproteins.

### 3.3. PT-1 Inhibited gp41 Core Formation and gp120-CD4 Interaction

**Figure 3:**
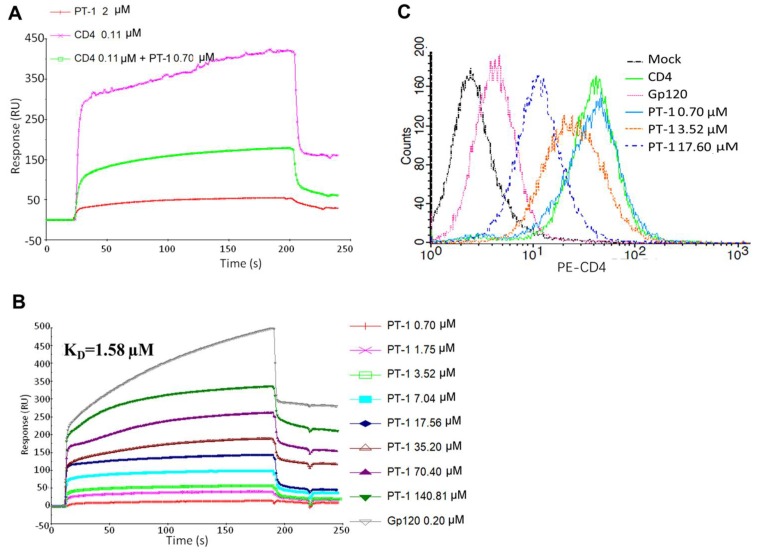
PT-1 inhibited gp120/CD4 binding. (**A**) Surface plasmon resonance (SPR) signals of gp120/CD4 binding. Recombinant gp120 protein was immobilized on a CM5 sensor chip, followed by injection of soluble CD4 (0.11 μM), PT-1 (2 μM), or a pre-incubated mixture of CD4 (0.11 μM) and PT-1 (0.7 μM); (**B**) SPR sensorgram of PT-1 binding to CD4 binding. CD4 protein was immobilized on a CM5 sensor chip, followed by injection of a serial dilution of PT-1 (0.7–140.81 μM). Gp120 was used as a positive control; (**C**) FACS profiles of PT-1 binding to the CD4 receptor on MT4 cells. MT4 cells were incubated PT-1 (0.7, 3.5, 17.6 μM) or gp120 (positive control) for 30 min. Cells were then stained with PE-conjugated anti-CD4 RPA-T4 antibodies (D1 domain) for FACS analysis.

The surface plasmon resonance-based competition assay was performed by immobilizing gp120 recombinant protein to react with recombinant soluble CD4 protein in solution. We noted that pre-incubation of CD4 with PT-1 caused a substantial reduction in the binding signal, whereas PT-1 did not bind directly to gp120 itself, suggesting that PT-1 competed with gp120 for binding to CD4 (Figure 3A). We then analyzed the binding of PT-1 to CD4 recombinant protein using a direct binding assay, where CD4 protein was immobilized on a CM5 chip, and PT-1 was run in a serial of dilutions. The average K_D_ value for the PT-1-CD4 interaction was 1.58 µM using a 1:1 langmuir binding model (Figure 3B). To further elucidate the binding ability of PT-1 to the natural CD4 receptor, we co-incubated PT-1 with MT4 cells and stained the cells with anti-CD4 RPA-T4 antibodies, which bind to the D1 domain (gp120 binding domain) of CD4. FACS analysis revealed that PT-1 suppressed the fluorescence intensity in a concentration-dependent manner (Figure 3C)., In addition, PT-1 incubation did not induce IL-2 secretion (Figure S3), suggesting that PT-1 may interfere with the gp120 binding site on the CD4 receptor without activating CD4 T cells. We also compared the effects of PT-1 on CCR5-(YU2) and CXCR4-(NL4-3) utilizing strains; however, no differences were observed. PT-1 also did not inhibit the binding of specific anti-CXCR4 and anti-CCR5 monoclonal antibodies to MT4 cells (data now shown), suggesting that PT-1 did not interact with any of the co-receptors.

**Figure 4:**
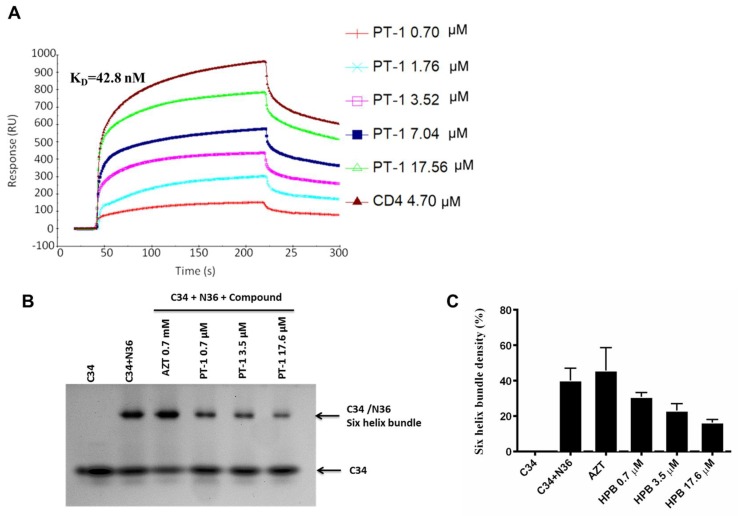
PT-1 inhibited HIV gp416-HB formation. (**A**) SPR sensorgram of PT-1 binding to the gp41 N36 peptide. N36 peptide was immobilized on CM5 sensor chips. PT-1 was injected over the surface at concentrations from 0.7 to 17.6 Nm; (**B**) N-PAGE analysis of the inhibitory effects of PT-1 on gp41 6-HPB formation by N36 and C34 peptides. Lane 1: C34, lane 2: C34 + N36, lane 3: C34 + N36 + AZT (0.7 mM, negative control), lane 4: C34 + N36 + PT-1 (0.7 μM), lane 5: C34 + N36 + PT-1 (3.5 μM), lane 6: C34 + N36 + PT-1 (17.6 μM). The average relative density of C34+N36 bands of each lane were analyzed (**C**). All results were obtained from three independent experiments.

We also performed native polyacrylamide gel electrophoresis (N-PAGE) to detect the effects of PT-1 on gp41 6-HB formation, as shown by N-PAGE (Figure 4B,C). N36 alone showed no bands because of the net positive charge, whereas C34 alone showed a clear band at a low position in the gel (Lane 1). When N36 was mixed with C34, a new band corresponding to the 6-HB formed by N36 and C34 appeared at the higher position (Lane 2). After incubation with PT-1, the density of the 6-HB band was weakened, and recovery of the C34 band was observed (Lanes 4–6). In addition, AZT did not reduce 6-HB formation (Lane 3). These data indicated that PT-1 affected the functional conformation of 6-HB. Similarly, PT-1 showed clear concentration-dependent binding affinity with the N36 peptide in SPR assays, with PT-1 bound to immobilized N36 showing an average K_D_ of 42.8 nM (Figure 4A). Taken together, these data revealed that PT-1 inhibited HIV-1 Env-mediated entry by blocking the gp120 binding site of the CD4 receptor and interacting with the viral gp41 NHR.

### 3.4. PT-1 Showed Modest Inhibitory Activity on Viral Reverse Transcriptase and Integrase

**Figure 5:**
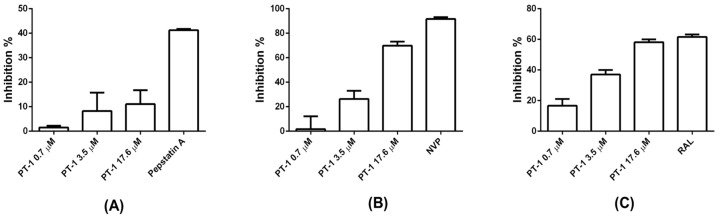
Effects of PT-1 on HIV-1 reverse protease, transcriptase and integrase 3′ processing activity. (**A**) Protease activity was assessed using a commercial kit, and pepstatin A was used as a positive control; (**B**) Reverse transcriptase activity was analyzed using a commercial kit with colorimetric assays; the percent inhibition was calculated as compared with the reverse transcriptase control. Nevirapine (NVP, 5 μM) was included as a positive control; (**C**) Integrase 3′-processing activity was analyzed by fluorescence resonance energy transfer. RAL was used as a positive control. All data were means ± standard deviations (SD) and were representative of three independent experiments.

We further examined the ability of PT-1 to inhibit the viral protease, reverse transcriptase and integrase. Pepstatin A, nevirapine (NVP), and RAL were used as positive controls, respectively. PT-1 did not suppress protease activity (Figure 5A) but blocked reverse transcriptase activity (Figure 5B) and HIV-1 integrase 3′ processing (Figure 5C) only at the highest concentration (17.6 μM). The maximum inhibition rates of reverse transcriptase and integrase 3′ processing were approximately 70% and 60%, respectively. These data implied that in vitro reduction of reverse transcriptase and integrase activity was unlikely to be major mechanism of PT-1 induced HIV-1 inhibition at nanomolar concentrations.

## 4. Discussion

The antiviral activity of POMs was first documented in 1970s, and they were later extensively studied in influenza, polioviruses, herpes simplex virus, etc. [13,14,15]. Especially, most POMs were found effective against HIV-1, HIV-2 and simian immunodeficiency virus (SIV) [21,35]. HPA-23, known as antimonium tungstate, was one of the first-generation POM and the only one of all studied POMs so far that progressed to clinical trials. However, it caused adverse effects on the hepatic and renal functions [36]. POMs still suffer from the stigma of this failure, although new generations of POMs including Keggin structures, have been synthesized with less toxicity and greater efficacy, most studies have focused on cancer therapy [37,38], and very few studies have been conducted to investigate the anti-HIV potential in the past decade [39]. The mechanism of action has remained unclear. In the present work, we evaluated the potency and mechanism of action of a Keggin POM, PT-1 (K_6_HPTi_2_W_10_O_40_) in preventing HIV-1 entry and replication. We demonstrated that PT-1 inhibited replication of multiple virus strains with IC_50_ values in the nanomolar range. In addition to its remarkable high potency, it revealed low cytotoxicity and genotoxicity. The high therapeutic index (TI) makes PT-1 a good candidate for HIV-1 treatment.

Although the antiviral activity of POMs has been documented, the mode of antiviral action has remained elusive. Previous studies have demonstrated that POMs could block the HIV-1 reverse transcriptase (RT) or protease [19,40]. Thus, we conducted the assay to test the inhibition on RT, protease, and integrase. PT-1 exhibited a certain degree of inhibition on RT and integrase, but not protease. We also observed that the RT inhibition percentage was not comparable to the HIV-1 inhibition percentage, indicating that the anti-HIV-1 activity was not fully mediated via inhibition of RT or integrase activity. To learn more about which steps of the HIV-1 cycle were targeted by PT-1, we conducted a time-drug-addition assay to have a thorough investigation of possible targets of the HIV-1 life-cycle. By adding PT-1 at different time points, we showed that PT-1 exhibited inhibition at a very early step of virus replication. Moreover, PT-1 specifically interacted with the HIV-1 envelope and not with VSV-G, thus the viral entry process might be the most likely antiviral target of PT-1. 

The HIV-1 envelope glycoproteins, composed of gp120 and gp41, are responsible for virus binding and membrane fusion. The first step in the HIV-1 entry into the host cells involves the binding of the gp120 envelope glycoprotein to the CD4 on the target cell membrane. Previous studies revealed that POMs were effective in blocking viral absorption by interacting with the gp120 glycoprotein [41]. In our assays, PT-1 did not directly bind to gp120 in the SPR assay, however, it competed with CD4 for binding to gp120. The CD4 receptor is a monomeric protein composed of four immunoglobulin (Ig)-like extracellular domains (D1-D4). Numerous mutagenesis tests indicated that the D1 domain (complementarity-determining region (CDR2, region 21–64) is critical for gp120 binding and can be blocked by anti-CD4 antibody [42,43]. We thus used anti-CD4 RPA-T4 antibody (binds to D1 domain) to analyze the interaction of PT-1 and CD4 on the cell membrane. The FACS analysis confirmed the direct binding of PT-1 to the D1 domain of the CD4 receptor. When gp120 interacted with CD4 and co-receptor CXCR4 or CCR5, the gp41 transmembrane subunit experiences conformational changes, and the N-terminal heptad repeats (NHR) of gp41 associate with the C-terminal heptad repeat (CHR) to form a thermostable, six-helix bundle (6-HB) core. The NHR and CHR domains are critical for the membrane fusion. NHR and CHR derived protein, such as N36 and C34, blocked gp41 core formation by interacting with CHR and NHR, respectively, and served as important targets for HIV fusion inhibitors [44]. In our study, PT-1 interfered with gp41 formation by binding to an NHR peptide. The K_D_ value of PT-1 binding to N36 was close to the IC_50_ obtained in the cell-based assay, suggesting that PT-1 inhibited HIV-1 entry by interfering with NHR and disrupting gp41 six helix bundle formation.

The current antiretroviral therapy is a combination of three or more antiretroviral drugs that target different steps of HIV-1 life cycle. Despite the fact that it reduces the viral load to an undetectable level and has greatly improved survival rates and life quality of HIV-infected patients, it still faces challenges including long-term drug resistance, unwanted drug-drug interactions and low compliance of patients. Thus, the discovery of multifunctional agents that interact simultaneously with more than one target has proven effective in anti-HIV leads discovery. For instance, inducible T cell kinase (ITK) and mammalian target of rapamycin (mTOR) inhibitors blocked multi-steps of HIV replication, including viral entry, reverse transcription, integration, and viral particle production [45,46]. Many plant-derived polyphenols such as quercetin, isoflavones, flavanones and phenolic acid derivatives, showed activity against various viral enzyme targets simultaneously [47]. Importantly, HIV protease inhibitors were found not only affecting virion release, but also blocking viral entry, reverse transcription and post–reverse transcription [48]. In the present study, we have identified the multi-target profile of PT-1, which not only blocked viral entry, but also interfered with main HIV enzymes reverse transcriptase and integrase In addition, POMs could be easily modified by various chemical reactions to enhance the specificity or activity, and they are less expensive than organic agents. We synthesized a serial of POMs derivatives by decorating PT-1 with amino acid to improve biocompatibility, and the modifications did not affect anti-viral activity. Thus, PT-1 has shown some promising properties for drug development. It is also important to note that the pharmacokinetic and pharmacodynamics profile of most metal-based compounds have yet to be identified and warrant further investigation.

## 5. Conclusions

In summary, our results revealed a safe, potent and broad spectrum antiretroviral activity of PT-1 (K_6_HPTi_2_W_10_O_40_). The data suggested that PT-1 functioned during an early stage of infection, partially by directly interacting with the gp41 NHR and blocking the gp120-binding domain of the CD4 receptor. These findings suggest that PT-1 may be a promising drug candidate for the treatment of HIV-1.

## Figures and Tables

**Table 1:** In vitro antiviral activity and cytotoxicity of PT-1 against different HIV-1 strains.

HIV-1	Subtype	Cell Line	AZT	PT-1		
			EC_50_ (nM)	EC_50_ (nM)	CC_50_ (µM)	TI
TRO.11	B	TZM-bl	60.92 ± 36.93	5.26 ± 0.26	995.20 ± 106.07	1.89 × 10^5^
SC422661.8	B	TZM-bl	98.34 ± 11.68	16.69 ± 6.85		5.95 × 10^4^
REJO4541.67	B	TZM-bl	18.41 ± 11.23	1.96 ± 0.44		4.98 × 10^5^
Du422.1	C	TZM-bl	6.96 ± 2.73	484.56 ± 43.47		2.05 × 10^3^
CE1176	C	TZM-bl	28.23 ± 1.62	187.52 ± 22.46		5.31 × 10^3^
Q842env.d16	A	TZM-bl	11.45 ± 7.45	ND		ND
Q259env.w6	A	TZM-bl	24.77 ± 18.56	ND		ND
CNE8	AE	TZM-bl	30.73 ± 7.25	ND		ND
QA790.204I.ENV.A4	AD	TZM-bl	84.80 ± 42.73	62.48 ± 18.48		1.59 × 10^4^
QA790.204I.ENV.C1	AD	TZM-bl	2.43 ± 1.53	0.60 ± 0.25		1.65 × 10^6^
BJOX00200	CRF07_BC	TZM-bl	3.94 ± 0.91	ND		ND
CH119	CRF07_BC	TZM-bl	3.63 ± 1.01	237.16 ± 7.46		4.19 × 10^3^
HXB2	B	MT-4	128.11 ± 23.05	1248.32 ± 434.48		164.52
NL4-3	B	MT-4	101.00 ± 10.10	468.20 ± 112.37	205.37 ± 63.54	438.63
BH10	B	MT-4	68.03 ± 8.91	1566.51 ± 325.74		131.10

TI, therapeutic index. ND, not determined.

## Supplementary Materials

The following are available online at http://www.mdpi.com/1999-4915/10/5/265/s1, Table S1: Anti-HIV-1 activities of PT-1 in PBMCs from three donors. Table S2: Bone marrow micronucleus assay (x ± SD). Figure S1: Percentage of apoptotic cells. Figure S2: The anti-HIV activity of organic functionalized derivatives of PT-1. Figure S3: PT-1 did not induce IL-2 secretion.
