# Supplementary material for: Inhibition of Human Immunodeficiency Virus Type 1 Entry by a Keggin Polyoxometalate
Source: Viruses. 2018 May 16;10(5):265. doi: 10.3390/v10050265 (PMC5977258; doi:10.3390/v10050265)
Supplement: Supplementary file 1 [file viruses-10-00265-s001.pdf]

Table S1. Anti-HIV-1 activities of PT-1 in PBMCs from three donors

	D3		D5		D7		D9	
	EC_{50} (nM)	EC_{90} (nM)	EC_{50} (nM)	EC_{90} (nM)	EC_{50} (nM)	EC_{90} (nM)	EC_{50} (nM)	EC_{90} (nM)
Donor1	74.1	7530.3	47.52	5676.63	3.95	3716.26	1.84	3490.97
Donor2	15.45	3737.65	3.91	2146.26	1.66	1452.76	0.12	543.66
Donor3	66.07	6009.95	15	4059.36	4.03	2331.23	1.23	1237.89

Table S2. Bone marrow micronucleus assay (x ± SD)

Group (mg/kg)	Numbers	MNPCE/ 1000 PCEs	PCE/ (PCE+NCE)
Saline control	5	0.2±0.44	41.3±4.6
200	5	0.4±0.89	40.1±3.3
600	5	0.4±0.54	42.4±2.8
1800	5	0.2±0.44	41.7±3.4
CP control	5	16.2±11.6***	42.8±2.2

Abbreviations: CP, Cyclophosphamide, MN, Micronucleated, NCE, normochromatic erythrocytes, PCE, polychromatic erythroctyes, SD, Standard deviation

*** Significantly different from saline control at P< 0.001

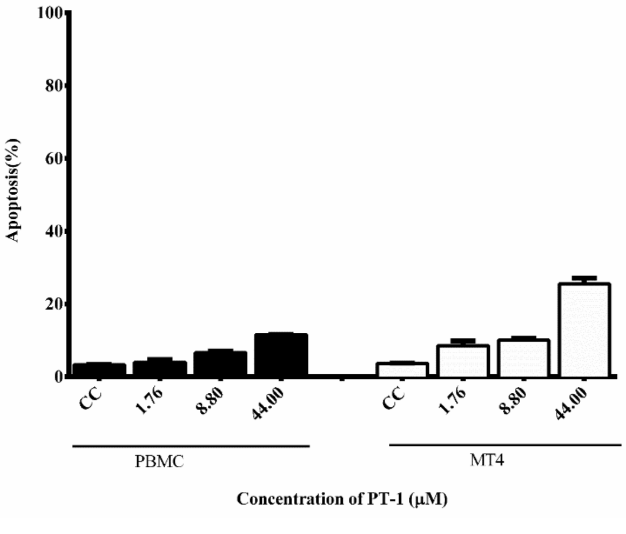

Figure S1 Percentage of apoptotic cell. MT4 T cells and pre-stimulated PBMCs were co-cultured with PT-1 (1.76-44.00 μM) for 5 days. Cell were then collected and stained with Annexin-V-FITC/PI. Cell apoptosis rates were analyzed by FACS. Results were expressed as mean ± SD.

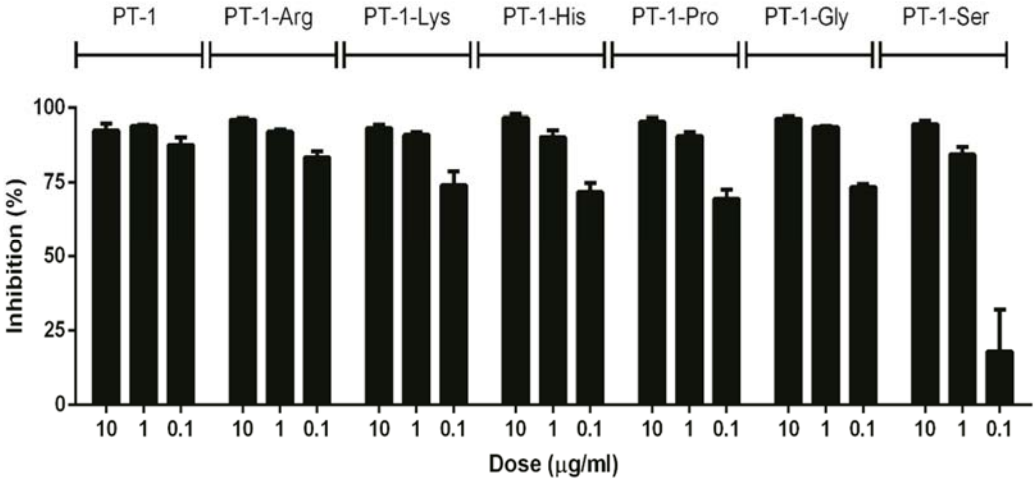

Figure S2. The anti-HIV activity of organic functionalized derivatives of PT-1. HIV-1 replication were evaluated by TZM-bl assays. PT-1 derivatives include Argnine (PT-1-Arg), Lysine (PT-1-Lys), Histidine (PT-1-His), Proline (PT-1-Pro), Glycine (PT-1-Gly) and Serine (PT-1-Ser) modified side-chains. The data were expressed mean + SD.

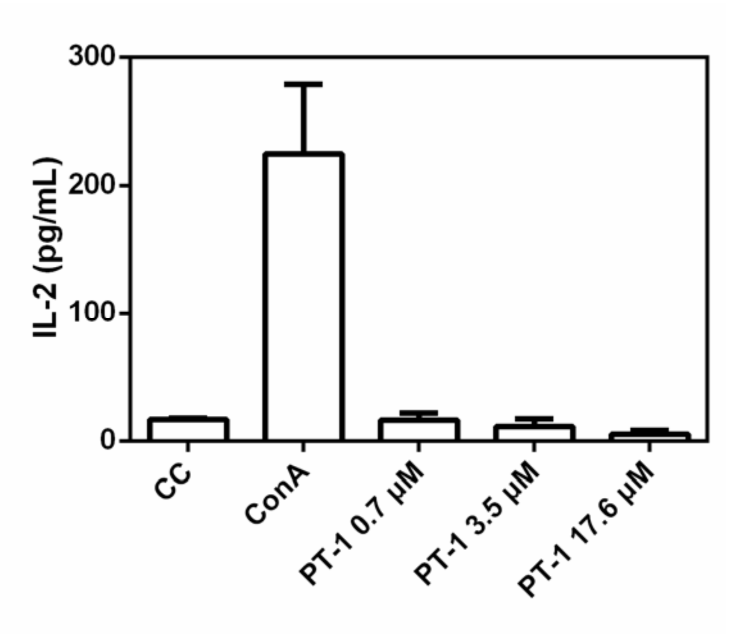

Figure S3 PT-1 didn't induce IL-2 secretion. PBMCs were isolated and co-cultured with PT-1 (0.7, 3.5, 17.6 μM) for 2 days. ConA (5 μg/mL) served as positive control. The supernatant were collected and measured for IL-2 secretion by ELISA. Data were expressed mean + SD.
